# Molecular Expression Profile of Changes in Rat Acute Spinal Cord Injury

**DOI:** 10.3389/fncel.2021.720271

**Published:** 2021-09-30

**Authors:** Jun-Juan Wang, Guo Ye, Hao Ren, Cheng-Rui An, Lvxing Huang, Hengyi Chen, Hui Zhang, Jun-Xin Lin, Xilin Shen, Boon Chin Heng, Jing Zhou

**Affiliations:** ^1^Dr Li Dak Sum & Yip Yio Chin Center for Stem Cells and Regenerative Medicine and Department of Orthopedic Surgery of The Second Affiliated Hospital, Zhejiang University School of Medicine, Hangzhou, China; ^2^School of Basic Medical Sciences and Forensic Medicine, Hangzhou Medical College, Hangzhou, China; ^3^Zhejiang University-University of Edinburgh Institute, Zhejiang University School of Medicine and Key Laboratory of Tissue Engineering & Regenerative Medicine of Zhejiang Province Zhejiang University School of Medicine, Hangzhou, China; ^4^Shenzhen Institute for Innovation and Translational Medicine, Shenzhen, China; ^5^Shenzhen ChanGene Biomedicine Technology Co. Ltd, Shenzhen, China; ^6^Peking University of School of Stomatology, Beijing, China; ^7^China Orthopedic Regenerative Medicine Group (CORMed), Hangzhou, China

**Keywords:** spinal cord injury, single cell sequencing, protein microarrays, differentially expressed genes, inflammatory factors

## Abstract

**Background:** Spinal cord injury (SCI) is a highly lethal and debilitating disease with a variety of etiologies. To date, there is no effective therapeutic modality for a complete cure. The pathological mechanisms of spinal cord injury at the molecular gene and protein expression levels remain unclear.

**Methods:** This study used single-cell transcriptomic analysis and protein microarray analysis to analyzes changes in the gene expression profiles of cells and secretion of inflammatory factors respectively, around the lesion site in a rat SCI model.

**Results:** Single-cell transcriptomic analysis found that three types of glial cells (microglia, astrocyte, and oligodendrocyte) becomes activated after acute injury, with GO exhibiting a variety of inflammatory-related terms after injury, such as metabolic processes, immune regulation, and antigen presentation. Protein microarray results showed that the levels of four inflammatory cytokines favoring SCI repair decreased while the levels of nine inflammatory cytokines hindering SCI repair increased after injury.

**Conclusion:** These findings thus reveal the changes in cellular state from homeostatic to reactive cell type after SCI, which contribute to understand the pathology process of SCI, and the potential relationship between glial cells and inflammatory factors after SCI, and provides new theoretical foundation for further elucidating the molecular mechanisms of secondary SCI.

## Introduction

Spinal cord injury (SCI) is a severe traumatic injury that usually causes loss of movement and sensory function in the limbs, eventually ending in paralysis. It has been estimated that there are over 300,000 patients suffering from SCI, with approximately 12,000 new cases occurring every year in the United States alone (Devivo, 2012). This not only causes much morbidity to patients, but also significantly reduces their life expectancy (J. Spinal Cord Med, 2016). Despite great advances in conservative therapies for SCI, there are still no effective treatment modalities for such devastating injuries due to the unclear and complex pathological process that take place after SCI. Therefore, it is imperative to explore the underlying molecular mechanisms in SCI to develop better therapeutic strategies.

### The Pathological Events After Spinal Cord Injury Include Two Processes

primary injury and secondary injury (Mcdonald and Sadowsky, 2002). Primary injury is the direct result of mechanical trauma on the spinal cord, which is an irreversible acute injury process that is extremely difficult to treat. However, the secondary injury triggered by primary injury is a reversible and controllable process, which could be relieved or mitigated, so as to alleviate symptoms and reduce further complications of SCI (Shi et al., 2017). Hence, it is critical to understand how the secondary injury aggravate the course of SCI and explore the underlying mechanisms in pathophysiology. Current research has indicated that secondary injury occurs within minutes to hours after SCI and leads to a series of detrimental processes around the lesion area, including inflammatory cell infiltration, neuronal apoptosis, glial scar formation, and oxidative stress (Yin et al., 2012; Fan et al., 2018).

In recent years, an increasing number of studies have shown that aberrant expression of multiple genes and activation of inflammatory responses are implicated in the pathological process of SCI (Mcdonald and Sadowsky, 2002; Ren et al., 2018). With the rapid development of high-throughput RNA sequencing technology, this has been widely applied in biomedical research to systematically investigate the molecular mechanisms of various disease pathologies. Therefore, this study aimed to systematically study the molecular mechanisms of pathology after secondary spinal cord injury. Comparative changes in the expression levels of various inflammatory factor and other key genes, before and after SCI in rats, will be analyzed by single-cell sequencing and protein microarray technology, to reveal the pathophysiological changes after SCI.

## Materials and Methods

### Animal Models

Adult female Sprague Dawley (SD) rats (250–300 g, *n* = 18) were purchased and housed at the Center for Experimental Animals, Zhejiang University at constant room temperature. All animal procedures complied with the Guidelines for the Care and Use of Laboratory Animals and were approved by the Animal Care and Use Committee of Zhejiang University. The animals were randomly assigned into the uninjured group (*n* = 6) and SCI group (*n* = 12). To perform the contusive SCI, the rats were anesthetized with pentobarbital (50 mg/kg) and received a T9-T10 vertebral laminectomy. After exposure of the spinal cord, a complete right hemi-section was performed at T10 with microscissors, resulting in an acute injury to the spinal cord at T10. After injury, the muscles and connective tissues were sutured, and the incision was closed with wound clips, with the animals being returned to their home cages. Rats in the uninjured group only received a laminectomy without spinal cord transection injury. Additionally, 80,000 units of penicillin were injected intramuscularly once a day for 3 consecutive days to prevent infections. Animals were culled at different time intervals post-injury. For the uninjured group, samples were collected immediately after laminectomy surgery, while in the injury group, samples were collected from rats at 1, 7, and 21 days post-injury (dpi) respectively for corresponding experiments.

### Behavioral Assessment

We used the Basso-Beattie-Bresnahan (BBB) locomotor rating scale to assess the functional recovery of hindlimbs after SCI. For the test, rats were placed in an open field (80 × 130 × 30 cm) and covered with a non-slip cardboard floor for 4 min. At each detection stage, two examiners who were completely blinded to the treatment of the animals separately observed the animals and analyzed them at 0, 1, 7, 14, and 21 days post-injury. The full score of the BBB scale is 21 points. This scale is designed to assess the recovery of hind limb movement after thoracic spinal cord injury.

The grid-walking test has been recognized as an evaluation of sensorimotor function. The animals were placed on an elevated plastic-coated wire mesh (40 × 45 cm, 2 cm^2^ grid spaces) to walk, and the paw placement of their hind limbs was evaluated at the same time. The placement lasted for 3 min, allowing animals to walk freely on the platform. In this process, “misstep” is defined as the animal’s entire foot passing through the grid. Finally, the total number of steps walked and the percentage of misssteps of each injured limb were scored. For each experimental animal, such a grid-walking test was carried out at 0, 1, 7,14, and 21 days post-injury.

### Single-Cell RNA-Seq

#### Preparation of Single-Cell Suspension

A segment of spinal cord with a length of 5 mm centered on the injury point was dissected, which was 2.5 mm above and below the injury point. For the single-cell RNA-seq experiments, the tissue of the spinal cord of the uninjured group (*n* = 3) and acute SCI group (1dpi) (i = 3) were washed, and then chopped by small scissors within a droplet of Dulbecco’s modified Eagle’s medium (DMEM), followed by digestion in 0.05% (w/v) trypsin (Life Technologies) diluted in low-glucose DMEM (L-DMEM) (Gibco) solution at 37°C for 10 min. Then, the tissues were left to settle down and the supernatant was collected in a fresh 50 mL tube, before adding 10% (v/v) of fetal bovine serum to inactivate the trypsin. The tissue was then agitated to obtain the cell supernatant. The supernatant was collected in a fresh 50 mL tube, and filtered through 70 μm cell filters (Falcon BD). This was followed by centrifugation for 10 min at 1600 rpm, and re-suspending the pellet in DMEM containing 2% (v/v) serum.

#### Single-Cell Capture, cDNA Library Preparation, and Sequencing

Single cell capture, RNA extraction, and cDNA preparation were performed following the methods described in the Fluidigm protocol (PN 1009886, Using the C1 HT IFC to Generate Single-Cell cDNA Libraries for mRNA Sequencing). Briefly, cells were loaded onto the protein chip at a concentration of 300-500 cells/uL, imaged and assessed by phase-contrast microscopy to exclude wells with more than one cell. cDNA libraries were prepared on the Fluidigm C1 system (PN 1009886, C1 HT IFC) according to the manufacturer’s protocol. The cDNA reaction products were quantified with Qubit and then diluted with C1 Harvest Reagent to a final concentration of 0.2 ng/μL. The diluted cDNA reaction products were then converted into mRNA-seq libraries using the Nextera XT DNA sample preparation kit (Illumina, FC-131-1096, -2001 and -2002) following the manufacturer’s instructions. Sequencing of libraries was performed using the Illumina HiSeq X Ten sequencing platform.

#### Processing of the scRNA-Seq Data

The obtained clean data was mapped to the rat genome with Bowtie2 using default parameters. Reads count for each gene in each sample was counted by HTSeq. The reads counts matrix were quality controlled and normalized with procedures in MATLAB, as follows: 1. Remove all non-protein coding genes; 2. Remove all ribosomal protein genes. 3, Normalize data with CPM; 4, Remove all cells with mitochondrial genome fragments greater than 50%; 5, Remove all features if they did not have CPM > 2 in at least 2 samples undergoing the procedure above. 6, Remove samples from empty wells according to the relationship between number of Reads and Features (Supplementary Figure 1). Finally, 131 cells and 8199 genes remained for further analysis. Dimension reduction, clustering, and differential gene expression were analyzed in Seurat. Dimensional reduction was performed and cell clusters were identified based on the most significant principle components. Marker genes enriched in each cell cluster were identified using the default algorithm in Seurat. Differently expressed genes (DEGs) were defined as P-value < 0.05. Functional annotation of the resulting marker gene lists relative to GO terms was performed using the DAVID website.

### Protein Isolation and Multiplex Immunoassay

The spinal cord segment is the same as described in section “Behavioral Assessment,” which is a segment of the spinal cord in which a length of 5 mm centered on the injury point was selected, that was 2.5 mm above and below the injury point. Spinal cords (T9-T10 spinal cord segment containing the injury epicenter) of sham, acute, subacute, and chronic phases were harvested at 0, 1, 7, and 21 days after injury, respectively (*n* = 3 in every group). Tissues were isolated from the T9-T10 injury epicenter and homogenized with a razor blade in cold phosphate buffered saline (PBS). After centrifugation (6000 rpm, 15 min), the supernatant thus obtained was used for immunoassay. Protein samples were checked to have sufficient concentration and purity according to the manufacturer’s instructions. The concentrations of inflammatory factors in the supernatant were analyzed by a commercial Luminex immunoassay kit (Cytokine & Chemokine 22-Plex Rat ProcartaPlex^TM^ Panel, Thermo Fisher Scientific). For the 22 proteins in the Cytokine & Chemokine 22-Plex Rat ProcartaPlex Panel, the standard curve of each protein had been checked whether it meets the requirements specified by the manufacturer’s instructions. All detailed procedures were carried out according to the manufacturer’s instructions. The normalization of data analysis was set to the average value of day 0 to 0.

### Statistics Analyses

All quantitative data was analyzed with Kruskal-wallis analysis. The *post hoc* test of significant difference between groups were done according to Tukey’s honest significant difference criterion. Statistic analysis of GO followed the instruction of Clusterprofiler with default parameters. Values of *p* < 0.05 were considered to be significantly different.

## Results

### Hemisection Spinal Cord Injury Model in Rats

**FIGURE 1:**
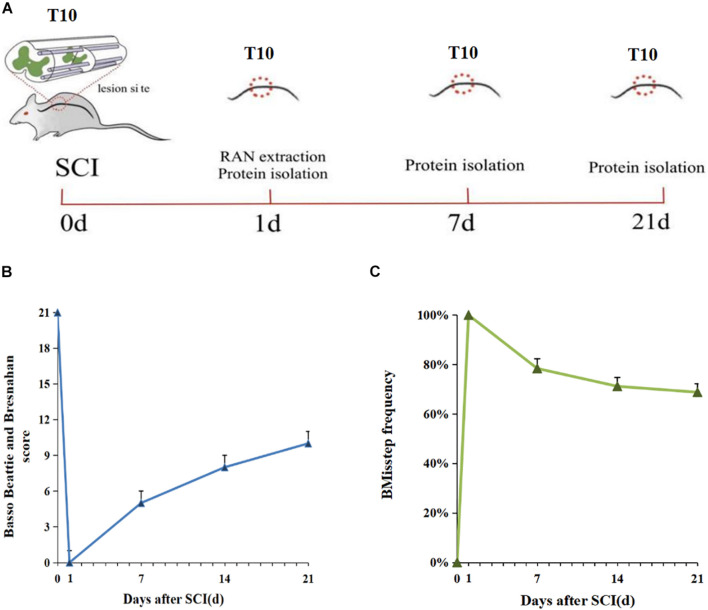
Schematic diagram of SCI and sample collection in the rat. **(A)** The red circles indicate the sites of axotomy and tissue sampling. **(B)** Weekly BBB (Basso, Beattie, and Bresnahan) score 0-21 days after SCI (*n* = 5). **(C)** Grid walking score 0–21 days after SCI (*n* = 5). The gait pattern characterization results of Grid walking were consistent with the BBB score.

We established a rat model of SCI by a complete right hemisection at the T10 position of the spinal cord (Figure 1A), and the right lower limb showed significant paralysis after surgery, which indicated successful creation of the model. BBB locomotor rating scale and grid-walking test were utilized to assess functional recovery at 0, 1, 7, 14, and 21 days post-injury. SCI caused severe debilitation, as indicated by dramatically decreased BBB scores and increased misstep frequency at 0, 1, 7, 4, and 21 days post-injury, as compared with the 0 day group. But all rats gradually displayed partial recovery of locomotor activity within days (Figures 1B,C). The above results were consistent with the results of gait pattern characterization.

### Molecular Identification of Oligodendrocytes, Astrocytes, and Microglia Cells at the Acute Injury Site

**FIGURE 2:**
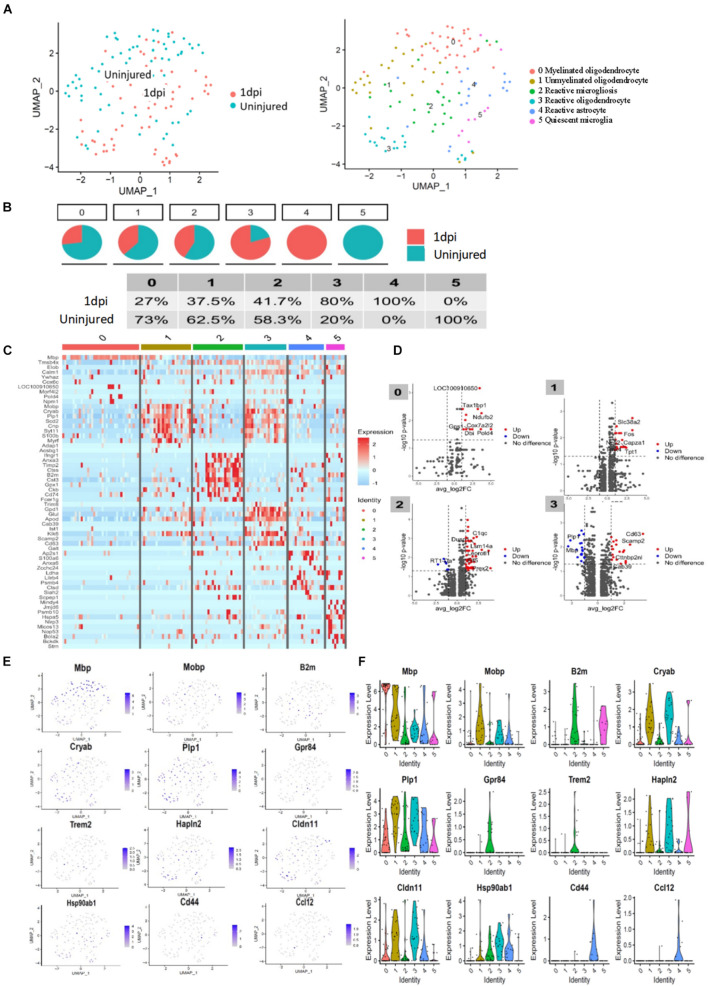
Transcriptomic identification of major cell clusters that comprise the rat uninjured group and 1 day post-injury group. **(A)** UMAP plot of all cells collected from the uninjured spinal cord and injured spinal cord at 1 day psot-injury. Cell clusters are colored and annotated based on a combination of DEGs, canonical marker genes, and previously published spinal cord scRNA-seq datasets. **(B)** Component of each cluster in the uninjured spinal cord and 1 day post-injury group. **(C)** Heatmap of genes were significantly enriched within each individual cluster. Single-cell in clusters are shown in columns; genes are shown in rows. Top ten differentially expressed genes of each cell type are shown. **(D)** Volcanic map of DEGs in uninjured spinal cord and 1 day post-injury group. **(E)** UMAP of expression of representative cluster-specific genes. Gray indicates low expression, while purple indicates high expression. Values indicated are log normalized counts per cell. **(F)** Violin plots showing distribution of expression for representative cluster-specific genes. Cell clusters are represented by different colors.

**FIGURE 3:**
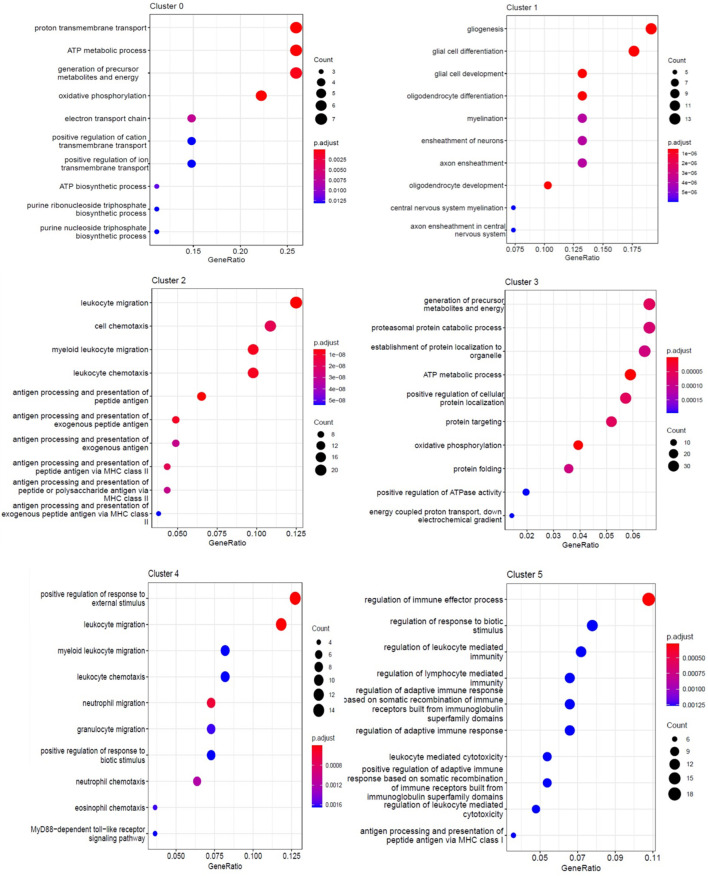
GO enrichment analysis of each cluster. The enriched GO terms (biological processes) of DEGs in each cluster.

To assess the cellular heterogeneity involved in spinal cord injury, we performed mid-thoracic hemisection SCI in rats and generated single-cell suspensions for sequencing. After excluding low-quality cells and potential doublets, we obtained a total of 131 cells from uninjured (69 cells from three animals), and 1 day post-injury (62 cells from three animals). Marker genes and injury-dependent genes offer useful information on cellular pathological states and can serve as potential biomarkers. Cluster analysis of these cells resulted in 6 distinct clusters when visualized on a uniform manifold approximation and projection (UMAP) plot (Figures 2A,B). Values of *p* < 0.05 were considered to be significantly different. All values of P shown in Supplementary Data Sheet 1 for analysis result. These 6 clusters represent three cell types that are known, including microglia, astrocytes, and oligodendrocytes. They were respectively group 0-myelinated oligodendrocytes cluster, group 1-unmyelinated oligodendrocytes, group 2-reactive microglia, group 3-reactive oligodendrocytes, group 4-reactive astrocytes, and group 5-quiescent microglia. Cells were grouped into these categories for further analysis as described below. Cell types pertaining to each cluster were identified using annotated lineage markers: myelinated oligodendrocyte (Mbp), unmyelinated oligodendrocyte (plp1+, Mbp−), reactive microglia (Gpr84, B2M, and Trem2), reactive oligodendrocyte (Hapln2, Cldn11, Hspa8, and Hsp90ab1), reactive astrocyte (Cd44, Slc16a3, and Ccl12), and quiescent microglia (RT1-CE10). Several injury-specific enriched genes and pathways are related to antigen presentation (B2M and CD74), and severe cell stress (Cryab, Hsp90ab1 Hspa8). When these markers are co-expressed with other cell-specific markers, this indicates that the cell type is in the activated state. Marker genes enriched in each cell cluster were identified using the default algorithm in Seurat. The heatmap showed single-cell data sets with clear differential gene expression modules and cell-type clusters (Figure 2C). The differentially expressed genes (DEGs,*P* < 0.05) provide a unique molecular signature for cluster1-4 (Figure 2D). Taken together, our analysis of DEGs between major cell types uncovers highly specific molecular identifiers, many of which are non-canonical (Figures 2E,F). Gene ontology (GO) analysis was conducted to gain a better functional insight on different cell clusters. The GO analysis results showed that the DEG of the 0 group cells were mainly involved in biological processes such as proton transmembrane transport, ATP metabolic process, etc. Group 1 were mainly involved in gliogenesis and glial cell differentiation, etc. Group 2 were mainly activated microglia, and their differentially expressed genes were mainly related to biological processes such as leukocyte mediated immunity and negative regulation of immune system processes. Group 3 were mainly related to biological processes such as ATP metabolism and oxidative phosphorylation. It is worth noting that we found that cells in group 5 (stable microglia) were mainly concentrated in the uninjured group. In addition, many items related to inflammation like antigen presentation and oxidative stress were highly expressed in cluster 2–4 (Figure 3). This information indicates that not only are microglia activated during the inflammatory process, but astrocytes and oligodendrocytes can also be activated in the acute SCI phase.

### Differentially Expressed Inflammatory Factors in Spinal Cords at Different Stages of Injury

**FIGURE 4:**
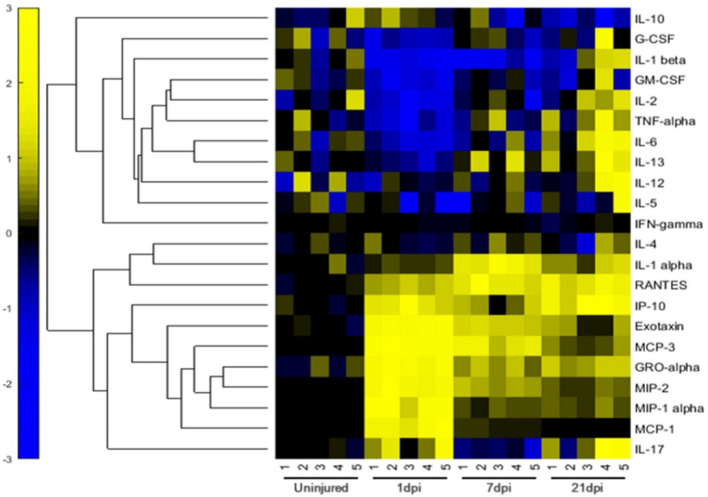
Relative Expression Levels of 22 inflammatory factors of rats in the 0-d group, 1-d group, 7-d group, and 21-d group after SCI. The normalization of data sets were based on the average value of day 0 to 0, yellow represents relatively high expression, while blue represents relatively low expression.

We evaluated the expression of 22 inflammatory factors after SCI, and found that different inflammatory factors were closely related to the pathophysiological process of SCI. The expression of inflammatory factors changes at different time points after injury. Among these, the expression levels of 10 inflammatory factors increased significantly after injury, while 8 inflammatory factors were significantly downregulated, while there was no change in the expression of 4 factors before and after injury (Figure 4). Values of *p* < 0.05 were considered to be significantly different. All values of P shown in Supplementary Data Sheet 2 for analysis result. We classified the inflammatory factors according to their discernible changes and whether they promoted or inhibited SCI repair (Table 1). Four inflammatory factors (GM-CSF, G-CSF, IL-10, and IL-13) that have been shown to promote recovery displayed consistently low expression at 1 and 7 days post-injury. IL13 expression gradually increased at 21 days post-injury. Nine inflammatory factors (Eotaxin, MCP-3, MIP-1α, MIP-2, GRO-α, IL-α, CXCL10, RANTES, and IL-17) have been shown to hinder SCI repair and were significantly upregulated at 1 day post-injury. Several factors (like MCP family) decrease at 21 days post-injury. No beneficial factors were elevated after injury. Therefore, the local environment after acute injury (1dpi-7dpi) is too harsh and unconducive for functional recovery.

**TABLE 1:** Classification of inflammatory factors of rats after SCI.

	Beneficial	Controversial	Detrimental	Unknown
Elevated		MCP-1	Eotaxin, MCP-3, MIP-1α, MIP-2, GRO-α, IL-α,CXCL10, RANTES, and IL-17	
Decreased	GM-CSF, G-CSF, IL-10, and IL-13	IL-2 and IL-6	IL-1β and TNF-α	
No change	IFN-γ, IL-12, and IL-4			IL-5

## Discussion

**FIGURE 5:**
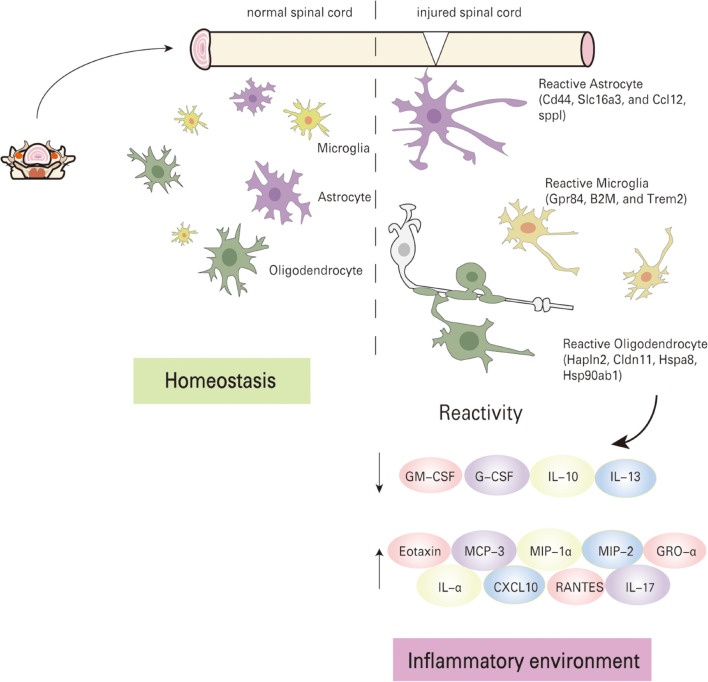
Schematic summary of molecular mechanism. The local pathological environment changes before and after spinal cord injury, the cells enter an activated state, and the inflammatory factors are seriously unbalanced.

The pathophysiological process of spinal cord injury is highly complex, and secondary injury after primary injury can further aggravate the extent of tissue damage. A series of complications often occur after SCI, in which changes in cellular gene expression levels and development of inflammatory responses play a significant role (Furlan and Fehlings, 2009). In this study, we analyzed changes in gene expression levels, as well as secretory levels of inflammatory factors before and after SCI by scRNA-seq and inflammatory factor immunoassays (protein chip) respectively. It was observed that microglia, astrocytes, and oligodendrocytes were activated at 1 day post-injury during the acute phase. Imbalance of pro-inflammatory and anti-inflammatory responses exacerbates the damage beyond repair (Figure 5).scRNA-seq has proven to be a robust technique for isolating and sequencing cells from fresh tissues, which do not have long processes (Voet et al., 2019; Schirmer et al., 2021). Thus microglia, astrocytes, and oligodendrocytes were easily collected for scRNA-seq analysis. Neurons were excluded from the analysis because they have long processes and larger volume that is not suitable for capture in our system. To date, there have not been many studies on cell heterogeneity after spinal cord injury. In this study, the Fluidigm C1 system was used in attempting to capture cells for analysis, but the small number of cells that were effectively captured placed a limitation on this analysis. Nevertheless, we can still draw some meaningful conclusions from the data.

Microglia are consistently tested in the peri-injury tissue after SCI and represents the major cellular contributors to post-SCI inflammation. The activities of microglia could be fine-tuned through the local microenvironment, and are ready to respond to local tissue injury. In our study, we found that microglia can increase their expression levels of co-stimulatory molecules (Apoe, lilrb4, csf1), and decrease expression of homeostatic microglial genes (Mertk, Tmem119). Antigen presentation (B2m and Cd74) can also be detected. GO analyses showed that these differentially expressed genes are mainly associated with antigen presentation after SCI in this group. Upon activation, microglia lose their homeostatic gene signature, undergo disease-specific changes, and release pro-inflammatory factors. These in turn lead to oxidative stress and cause nervous system tissue damage. The activation state was closely related to the severity of nerve damage. Microglia are responsible for antigen presentation at the normal state, express low levels of major histocompatibility complex molecules, but can rapidly be activated upon disease or injury. In the pathological state, microglia acquire signals secreted by the surrounding cells and change to a pro-inflammatory-phenotype immediately after SCI (Butovsky and Weiner, 2018). Astrocytes are the most abundant glial cell population within the mammalian CNS with highly specialized key roles in supporting neurons and pruning excitatory synapses. Various studies have shown that abnormal astrocyte activation is related to a variety of neurological diseases. GO results showed that this group of cells was mainly implicated in regulating various immune effector processes. In addition, Spp1 and Ccl12 are upregulated in the astrocyte group. It has also been documented that astrocytes can be activated through microglia or other immune cell subtypes after acute injury that eventually result in transcriptomic and morphological changes (Liddelow et al., 2017). These inflammatory cues may in turn induce astrocytes into a fully mature reactive state, which induces rapid death of neurons and oligodendrocytes (Faulkner et al., 2004). The reason that injured CNS would ever produce a neurotoxic reactive astrocyte is still a mystery. Drugs that prevent or revert these subtype of reactive astrocytes may save neurons after spinal cord injury and promote regeneration by stimulating growth and allowing these astrocytes to clear myelin debris (Fan et al., 2021). Oligodendrocytes also display high cell stress levels (Hapln2, Hspa8, and Hsp90ab1), with GO analysis showing many differentially regulated metabolic processes after injury, which indicates that oligodendrocytes transit from a normal to active state. Meanwhile, the Mbp violin graph shows Mbp expression during the process of rapid loss after acute injury from Group 0 to Group1. These results suggest that oligodendrocytes undergo changes between morphological subtypes after injury, and are also involved in the development of inflammation. Therefore, reactive oligodendrocytes are not just involved in pathological changes after injury, but are also active players in SCI inflammation progression. These observed behavior of oligodendrocytes after injury have many similarities to that seen in multiple sclerosis of the spinal cord (Jäkel et al., 2019). After early injury, various cells undergo transition from the normal state to stressed state, while at the same time, a large number of inflammatory factors are released into the local environment (Liddelow et al., 2017). We systematically detected changes in the expression levels of inflammatory factors before and after SCI via protein chip technology. Our results showed the trend in the expression of 22 inflammatory cytokines over time. Inflammatory factors that have been shown to hinder SCI repair were upregulated significantly at 1 day post-injury. Most inflammatory factors (IL-α, RANTES, IP-10, and GRO-α) display high expression during 21 days post-injury. The secretory levels of several factors (MCP-3, MIP-2, and MIP-1α) decrease after 21 days. Some inflammatory factors are beneficial to the repair of SCI. In our study, four factors (GM-CSF, G-CSF, IL-10, and IL-13) that have been shown to promote recovery displayed consistently low expression levels at 1 and 7 days post-injury. Only IL13 expression gradually increased at 21 days post-injury. GM-CSF has been reported to inhibit the formation of glial scars after SCI, enhancing the integrity of axon structure and producing long-term protection (Na et al., 2016). Moreover, GM-CSF and G-CSF can also determine the function of inflammatory macrophages by regulating glycolysis and lipid metabolism signaling pathways, thereby exerting anti-inflammatory effects (Na et al., 2018). IL-10 and IL-13 are stimulatory factors for M2 macrophages. In SCI, they can abate the release of pro-inflammatory factors and reduce neuronal cell apoptosis, thereby promoting the functional recovery of SCI (Zhou et al., 2009; Li et al., 2014; Yang et al., 2016). The secretory levels of nine factors (Eotaxin, MCP-3, MIP-1, MIP-2, GRO-α, IL-α, CXCL10, RANTES, and IL-17) that have been shown to hinder SCI repair, were upregulated significantly at 1 day post-injury. These factors are usually secreted by a variety of neural lineage cells, such as neutrophils, monocyte, microglia, and astrocyte, etc. Activation of neutrophils, macrophages, lymphocytes, and microglia after SCI can all produce IL-17 (Kawanokuchi et al., 2008). The IL-17 signaling pathway further activates the NF-kB, MAPK, TNF α and other signaling pathways, which in turn induce cells to release pro-inflammatory factors that can inhibit or activate the expression of downstream inflammatory factors. Additionally, inflammatory factors can induce and promote the expression of HIF-1α in cells under hypoxic conditions. The overexpression of HIF will lead to a large production of inflammatory factors (Cramer and Johnson, 2003; Haddad and Harb, 2005; Karhausen et al., 2005). Therefore, after SCI, synergistic cross-talk between inflammatory signaling pathways further induces cascade amplification of inflammation, which plays a key role in the pathological process of SCI.

## Conclusion

In summary, we have comprehensively analyzed changes in the gene expression profiles of cells around lesion sites and secreted inflammatory factors in rats, before and after SCI by single-cell transcriptome sequencing and protein chip analyses respectively. We found that three types of glial cells (microglia, astrocyte, and oligodendrocyte) become activated after acute injury, as well as displayed significantly higher expression of inflammatory cytokines within the local environment after injury. These findings thus reveal the changes in cellular state from homeostatic to reactive cell type after SCI, which contribute to understand the pathology process of SCI, and the potential relationship between glial cells and inflammatory factors after SCI, and provides new theoretical foundation for further elucidating the molecular mechanisms of secondary SCI.

## Data Availability Statement

The data presented in the study are deposited in the Genome Sequence Archive in the National Genomics Data Center, China National Center for Bioinformation/Beijing Institute of Genomics, Chinese Academy of Sciences repository, accession number CRA004460.

## Ethics Statement

The animal study was reviewed and approved by the Animal Care and Use Committee of Zhejiang University.

## Author Contributions

JZ designed the study. HR and GY established the rat SCI model. C-RA and XS analyzed data. J-JW was a major contributor in writing the manuscript. J-XL drafted the work. LH, HC, BH, and HZ substantively revised the manuscript. All authors have read and approved the final manuscript.

## Conflict of Interest

HR was employed by the company Shenzhen ChanGene Biomedicine Technology Co. Ltd. The remaining authors declare that the research was conducted in the absence of any commercial or financial relationships that could be construed as a potential conflict of interest.

## Publisher’s Note

All claims expressed in this article are solely those of the authors and do not necessarily represent those of their affiliated organizations, or those of the publisher, the editors and the reviewers. Any product that may be evaluated in this article, or claim that may be made by its manufacturer, is not guaranteed or endorsed by the publisher.

## Abbreviations

BBB: Basso-Beattie-Bresnahan
BP: biological process
CC: cellular component
CXCL10: chemotactic ligand 10
DEGs: differential expression genes
Eotaxin: eosinophil chemotactic factor
GO: gene ontology
GRO-α: growth regulatory oncogene-α
HIF-1: hypoxia-inducible factor-1
IL-1: interleukin
IL-17: interleukin-17
KEGG: kyoto encyclopedia of genes and genomes
MCP-3: monocyte chemoattractant protein-3
MIP-2: macrophage inflammatory protein 2
MIP-1α: macrophage inflammatory protein 1α
MF: molecular function
RANTES: regulating and activating normal T cell
ESF: expression and secretion factor
SCI: spinal cord injury
SD: Sprague Dawley
TNF-α: tumor necrosis factor-α
UMAP: uniform manifold approximation and projection.
