# Supplementary figures and images for: Molecular Expression Profile of Changes in Rat Acute Spinal Cord Injury
Source: Front Cell Neurosci. 2021 Sep 30;15:720271. doi: 10.3389/fncel.2021.720271 (PMC8516027; doi:10.3389/fncel.2021.720271)

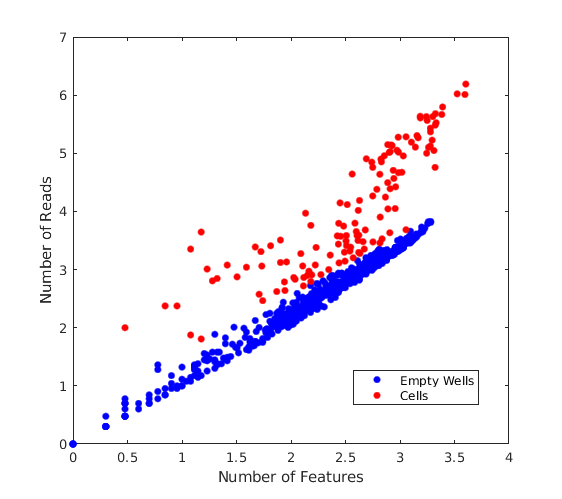

Supplement: Supplementary file 3 [file Image_1.tif]
